# Determinants of legacy effects in pine trees – implications from an irrigation‐stop experiment

**DOI:** 10.1111/nph.16582

**Published:** 2020-05-09

**Authors:** Roman Zweifel, Sophia Etzold, Frank Sterck, Arthur Gessler, Tommaso Anfodillo, Maurizio Mencuccini, Georg von Arx, Martina Lazzarin, Matthias Haeni, Linda Feichtinger, Katrin Meusburger, Simon Knuesel, Lorenz Walthert, Yann Salmon, Arun K. Bose, Leonie Schoenbeck, Christian Hug, Nicolas De Girardi, Arnaud Giuggiola, Marcus Schaub, Andreas Rigling

**Affiliations:** ^1^ Swiss Federal Institute for Forest, Snow and Landscape Research WSL 8903 Birmensdorf Switzerland; ^2^ Forest Ecology and Management Group Wageningen University 6701 Wageningen the Netherlands; ^3^ Institute of Terrestrial Ecosystems ETH Zurich 8092 Zurich Switzerland; ^4^ Dipartimento Territorio e Sistemi Agro‐Forestali University of Padova 35020 Legnaro Italy; ^5^ ICREA 08010 Barcelona Spain; ^6^ CREAF Universidad Autonoma de Barcelona 08193 Barcelona Spain; ^7^ Horticulture and Product Physiology Wageningen University Wageningen 6701 the Netherlands; ^8^ Institute for Atmospheric and Earth System Research/Physics University of Helsinki 00100 Helsinki Finland; ^9^ Institute for Atmospheric and Earth System Research/Forest Sciences University of Helsinki 00100 Helsinki Finland; ^10^ Forestry and Wood Technology Discipline Khulna University 9208 Khulna Bangladesh

**Keywords:** cambial activity, drought stress, ecological memory, irrigation experiment, osmoregulation, point dendrometer, radial stem growth, TreeNet

## Abstract

- Tree responses to altered water availability range from immediate (e.g. stomatal regulation) to delayed (e.g. crown size adjustment). The interplay of the different response times and processes, and their effects on long‐term whole‐tree performance, however, is hardly understood.
- Here we investigated legacy effects on structures and functions of mature Scots pine in a dry inner‐Alpine Swiss valley after stopping an 11‐yr lasting irrigation treatment. Measured ecophysiological time series were analysed and interpreted with a system‐analytic tree model.
- We found that the irrigation stop led to a cascade of downregulations of physiological and morphological processes with different response times. Biophysical processes responded within days, whereas needle and shoot lengths, crown transparency, and radial stem growth reached control levels after up to 4 yr only. Modelling suggested that organ and carbon reserve turnover rates play a key role for a tree’s responsiveness to environmental changes. Needle turnover rate was found to be most important to accurately model stem growth dynamics.
- We conclude that leaf area and its adjustment time to new conditions is the main determinant for radial stem growth of pine trees as the transpiring area needs to be supported by a proportional amount of sapwood, despite the growth‐inhibiting environmental conditions.

## Introduction

Physiological responses of plants in general, and trees in particular, are often explained by current environmental conditions in, for example, ecophysiological models (Steppe *et al.*, 2006; Zweifel *et al.*, 2007; Medlyn *et al.*, 2011), wood formation studies (Drew *et al.*, 2010; Rathgeber *et al.*, 2016; Delpierre *et al.*, 2019) or assessments of climate‐vegetation dynamics (Liu *et al.*, 2018; Ma *et al.*, 2019). However, a wide range of evidence documents the limitation of this common approach accounting for concurrent environmental drivers only and, instead, strongly suggests to additionally consider past conditions (Anderegg *et al.*, 2015; Ogle *et al.*, 2015; Jump *et al.*, 2017; Zweifel & Sterck, 2018; Kannenberg *et al.*, 2019a). This effect is commonly described with the term ‘legacy effect’ (Huang *et al.*, 2018; Peltier *et al.*, 2018) and is in this work used as a generic term encompassing various carry‐over or lagged effects (also called ecological memory effects). As a consequence, the stronger a legacy effect is, the lower becomes the predictive power of current conditions for a physiological response (Meinzer *et al.*, 2013; Jump *et al.*, 2017; Zweifel & Sterck, 2018). It is thus also a measure for the degree of decoupling of plant responses from the concurrent environmental conditions (Kannenberg *et al.*, 2019b; c).

There is fast‐growing statistical evidence for such legacy effects (Ogle *et al.*, 2015; Jiang *et al.*, 2019; Peltier & Ogle, 2019). Annual stem growth of trees, as an example, is generally not optimally explicable with current‐year conditions (*R* ^2^ = 20–40%) and including past conditions has been shown to result in greater fractions of explained variance (*R* ^2^ increased by 30% and more (Ogle *et al.*, 2015). But the question remains, how do legacy effects take place at a mechanistic, physiological level? By contrast with statistical evidence for the importance of legacy effects, little information is known about the potential mechanisms. Zweifel & Sterck (2018) recently proposed an approach considering the turnover rates of organs and reserves as a way to link past conditions to the current plant response. They employed a model showing that differences in turnover rates can theoretically explain different levels of responsiveness to current conditions. Trees with generally longer turnover rates of leaves, sapwood and carbon reserves (> 5 yr) were found to be less responsive to a sudden change in the environmental conditions than trees with shorter organ turnover rates of 1 or 2 yr. It means that the former type of trees is better able to buffer short‐term negative environmental impacts. However, it also means that it takes longer to recover from severe impacts, in terms of re‐building structures of the predisturbance level (Zweifel *et al.*, 2000; Weber *et al.*, 2007; Galiano *et al.*, 2011; Zweifel & Sterck, 2018).

The turnover rate of an organ or a reserve is defined as the time in which the underlying structure (e.g. the needles of the crown, or the carbon molecules of the carbon reserve) is on average replaced. Consequently, the turnover rate of needles strongly affects the adjustment time of the total leaf area to changed conditions. For the sapwood, as another example, the turnover rate translates into the average number of years a tree‐ring remains part of the sapwood before it is turned into heartwood. Therefore, this individual tree‐ring with its specific number and distribution of water conducting elements and thus particular hydraulic properties, can affect the water conductance of a tree for a longer or a shorter time period depending on its functional life‐span. The value of such an organ and reserve turnover approach lies in its intrinsic capacity to mechanistically link past conditions to a current plant's response (Jump *et al.*, 2017; Zweifel & Sterck, 2018).

Here we test this idea of linking past and present environmental conditions to current physiological and morphological responses via organ and reserve turnover rates with field data from mature Scots pine trees which were experimentally irrigated since 2003 and cut off from the treatment after 11 yr at the end of 2013 (Dobbertin *et al.*, 2010; Schönbeck *et al.*, 2018; Brunner *et al.*, 2019). The setup of the Pfynwald irrigation experiment allows comparing control trees that were never irrigated with trees that received continued irrigation treatment until present, with trees for which irrigation was recently stopped (Bose *et al.*, 2020).

Scots pines in this dry inner‐Alpine valley (Valais) have been reported to suffer from increased mortality during the past decades (Zweifel *et al.*, 2009; Rigling *et al.*, 2013; Etzold *et al.*, 2019), a phenomenon caused by longer and more frequent dry periods (Bigler *et al.*, 2006; Szejner *et al.*, 2019). The irrigation treatment in the Pfynwald experiment added *c*. 500–600 mm water per year. This doubling of the natural precipitation increased stem radial growth, leaf area, needle and shoot lengths, whereas crown transparency decreased (Dobbertin *et al.*, 2010; Eilmann *et al.*, 2013; Schönbeck *et al.*, 2018). Furthermore, the irrigated trees adjusted their root production to the irrigation in terms of a significantly increased fine‐root biomass (Brunner *et al.*, 2009; 2019; Herzog *et al.*, 2014). Over the 11 yr of irrigation, these trees thus developed new (overbuilt) structures and adjusted their carbon reserves to the growth increase (Schönbeck *et al.*, 2018).

**Fig. 1:**
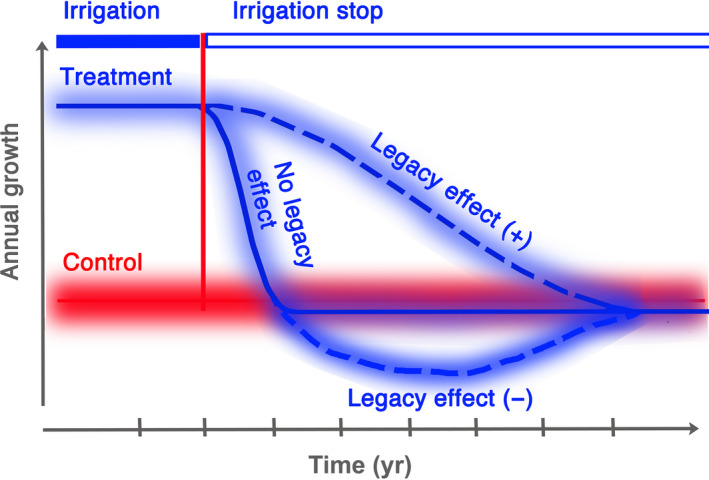
Alternative physiological or morphological responses of irrigated trees to a stop of the treatment in comparison with nonirrigated control trees. A positive legacy effect suggests that a response variable remains above the level of the control for several years. A tree without a legacy effect returns to the level of the control trees without delay. A negative legacy effect leads to a response variable below the level of the control before returning to the control level.

We propose three alternative response types of the trees growing in the irrigation‐stop plots (Fig. 1) in order to discuss timing and strength of legacy effects in different organs and the interplay among them at a whole‐tree level:

(**1**) In the first type, the physiological and morphological variables (i.e. sap flow, tree water deficit, stem growth, needle and shoot length, and crown transparency) return to the respective levels of the control trees in the first year after the irrigation stop. This type of response is not affected by past conditions (at least not to the extent as compared with the control) and is called here a type of response with ‘no legacy effect’.

(**2**) In the second type, the different variables are maintained above the level of the control for several years before returning to it. This response type is called a ‘positive legacy effect’ because past conditions induce a positive physiological or morphological response temporarily remaining above the level of the control. We hypothesise that such trees may benefit from reserves accumulated during the irrigated years.

(**3**) Finally, in the third type, the physiological responses drop below the control level before returning to it. This response is called ‘negative legacy effect’, as the past conditions alter the tree’s response in a negative way by shifting the process (temporarily) below the control level. This case reflects the idea that the ‘overbuilt irrigated trees’ are less well adjusted to the reduction in soil water after the irrigation stop than control trees and therefore perform worse.

## Materials and Methods

### Site

The long‐term irrigation experiment is located in a Scots pine forest (*Pinus sylvestris* L.) at the northwest‐exposed slope close to the bottom of the Swiss Rhone valley in the driest part of the Valais, (46°18′N, 7°36′E, 615 m asl) and close to the dry edge of distribution of Scots pine. Mean annual temperature was 9.2°C and mean annual precipitation 518 mm (1971–1990; Supporting Information Fig. S1) (Wehren *et al.*, 2010).

The forest is described as *Erico‐Pinetum sylvestris* with a mean tree height of 10.8 m, a stand density of 730 stems ha^−1^, and a basal area of 27.3 m^2^ ha^−1^ (Dobbertin *et al.*, 2010). A description of trees equipped with dendrometer and sap flow sensors can be found in Table S1. For the irrigated plots, precipitation has been approximately doubled since 2003 (additional 500–600 mm yr^−1^) by adding water from a nearby channel during night of the growing season (April–October) using sprinklers of 1 m height (Herzog *et al.*, 2014; Bose *et al.*, 2020). End of 2013, the irrigation was stopped in the upper third of each irrigated plot. The soil is a shallow Regosol characterised by low water retention capacity. All plots had the same exposition.

### General setup

We measured microclimatic variables in air and soil (data resolution: 10 min), physiological variables (10 min) and crown morphological variables (annual) on trees in the different subplots: control plots which never were irrigated (control), treated plots with irrigation until the end of 2013 (irrigation stop), and irrigated plots until the end of 2017 (irrigation).

**Fig. 2:**
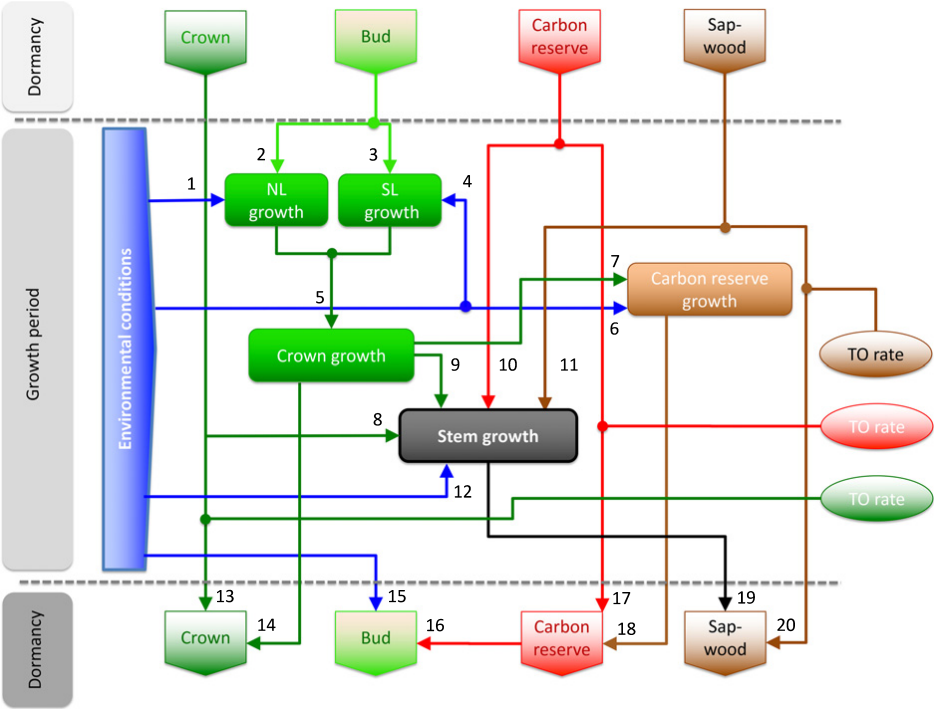
System‐analytical tree model to quantify legacy effects according to Zweifel & Sterck (2018). The model consists of four elements that describe the tree status at the beginning of the year (crown, bud, carbon reserve and sapwood). The model further takes up the key processes of radial stem growth, crown growth, needle length growth (NL), shoot length growth (SL), and carbon reserve growth. The elements are linked to a network with positive linear functions (indicated as arrows with numbers) weighted by a weighting factor (WF). Additionally, the turnover (TO) rates for the crown (needles), the sapwood and the carbon reserves quantify the time that is needed to renew the respective organ or reserve. With this network of functions, the new status of crown, bud, carbon reserve and sapwood are calculated. The model is run by an index for environmental conditions (Supporting Information Methods S2). Functions and additional explanations for the numbered arrows are given in Methods S1.

Additionally, we applied a system‐analytical tree model (Zweifel & Sterck, 2018) that bridges the influence of past conditions to the present physiological response on an annual level (Fig. 2). The model quantifies legacy effects on the organ and reserve status of a tree with the help of turnover rates of leaves, sapwood and carbon reserves. Those response variables, which were measured and modelled (radial stem growth, needle and shoot length), were compared in order to quantify the explanatory power of the ‘turnover approach’ to catch different patterns of legacy effects (Fig. 1).

### Environmental measurements

Meteorological measurements were recorded 2 m above the canopy on top of a scaffold, *c*. 13 m above ground. Air temperature (Sensirion, Stäfa, Switzerland), relative humidity (Sensirion) and precipitation (Young tipping bucket 52203, Michigan, USA) were continuously recorded at a 10 min interval. Data obtained in nearby meteorological stations of MeteoSwiss (www.meteoswiss.admin.ch) were employed to fill gaps and to correct obvious instrumental errors by applying filters and using simple regressions.

Soil water content was measured with time domain reflectometries (TDRs) (Tektronix 1502B cable tester, Beaverton, OR, USA) at soil depths of 10, 40 and 60 cm from 2002 to 2013. In spring 2014, the soil water measurement equipment was partially replaced and relocated. Since 2014, 10 HS‐Sensors (Decagon Devices, Pullman, WA, USA) were installed in all treatments and recorded soil water content at 10 and 80 cm soil depth. Soil water content data were gap‐filled and homogenised for the period from 2011 to 2017 with the help of overlapping periods of different soil measurement methods and devices. The logging devices used were from DecentLab (DecentLab GmbH, Dübendorf, Switzerland) and Campbell (CR1000, Logan, UT, USA).

### Physiological measurements

Stem radius (SR) changes were measured with point dendrometers at breast height (ZN11‐T‐WP; Natkon, Oetwil am See, Switzerland) on nine trees (three trees per treatment) consisting of a T‐shaped carbon fibre frame anchored in the stem with three stainless steel rods and a potentiometer. The dendrometers, including cables and loggers, have a low temperature sensitivity of < 0.3 µm per °C and SR data were not further corrected for temperature sensitivity. Furthermore, tree water deficit‐induced reversible stem shrinkage and swelling (TWD) and growth‐induced irreversible increment (GRO) were calculated with the R package treenetproc (Haeni *et al.*, 2020), according to the approach of Zweifel *et al*. (2016) assuming no cell growth during periods of stem shrinkage.

Stem sap flow was measured simultaneously with Granier‐type sensors (UP GmbH, Ibbenbüren, Germany) on the same trees where the dendrometers were mounted. The two needles of the sensors were drilled 5 cm into the sapwood and insulated from direct sunlight. Data resolution was 10 min. Sapwood depth was found to range between 23 and 107 mm in a sampling of 20 irrigated and 20 control trees in 2013 (other trees than the ones equipped with sap flow sensors). The mean sap wood width for control and irrigated trees was 47 and 51 mm, respectively (Fig. S2), found to be not significantly different (*t*‐test, *P* > 0.05). Sap flow was calculated from individual stem diameters and an average sapwood depth of 5 cm (370–425 cm^2^).

Sensors were powered and logged by devices establishing a local mesh network around a central base station with data transmission to the related online database (DecentLab).

### Crown morphology measurements

Needle and shoot lengths were measured from 36 trees (10 control, 12 irrigated, 14 irrigation stop). Three branches from each of these trees were selected from the top and the middle parts of the crown, resulting in 116 sampled branches. Shoot lengths on every branch were measured for the years 2011 to 2017 and averaged for the three treatments. Needle length of each shoot was determined by measuring five randomly selected needles close to the centre of the shoot.

Annual crown transparency was assessed by a visual rating in 5% steps of all (*c*. 800) trees of the experimental area using reference photographs ranging from 0% (a fully foliated tree) to 100% (a dead tree) as described by Dobbertin *et al*. (2005). The tree crown foliage is judged relative to the optimum foliage of an average tree of the same size and species. The average crown transparency of all trees within the different treatments (control, irrigation stop and irrigation) was used as a proxy for the development of the total leaf area.

### System‐analytical tree model

The model applied (Zweifel & Sterck, 2018) is based on physiological key processes such as crown growth, radial stem growth and carbon reserve growth (Fig. 2). The key processes described as linear functions (with weighting factors, Methods S1) are driven by environmental conditions (linearly coupled to tree water relations), and the status of crown, buds, carbon reserves and sapwood of the past year. We refer to this approach as a system‐analytic model (Vester, 2007) in which absolute mass or energy balances are not quantified, but the individual responses of system components are quantified relative to each other and relative to a ‘normal’ response (value = zero). This approach is particularly valuable to assess a system responsiveness, respectively the legacy effects of system parts (organs), in our case a tree.

The simulation in annual steps starts with the status of buds, crown, carbon reserve and sapwood that are the result of the past environmental conditions and the related processes over time. The initial status needs to be set before the first iteration. The length of time considered to affect the status of organs and reserves is defined by the turnover rates determining over how many years the respective structure is built and renewed, respectively. The model quantifies the current‐year environmental impact on all the processes involved and calculates the new status of the organs and reserves at the end of the year according to the network of functions (Fig. 2; Methods S1).

The model input is an annual environmental index calculated from water supply (precipitation plus irrigation), soil water content, air temperature, and radiation (Methods S2).

The model consists of linear functions with weighting factors (Methods S1) for which the input‐, operating‐ and output‐ranges are limited to index values ranging from −1 (very poor status), over 0 (average) to +1 (very good/improved status). This way, annual changes of environmental conditions (also expressed in values between −1 and +1) alter the status of structures (crown, bud, sapwood) and reserves (carbon reserve) according to the linear functions, their weighting factors and their turnover rates. Status values are thus always relative to an average value of zero. Values above zero always mean a status or a response above the average, a value below zero a status or response below the average.

The weighting factors (WF) for the linear functions between the status and the processes of the model were parameterised (Table S2) for the control trees (never irrigated, control), for the irrigated trees (irrigated), and for the trees treated with irrigation until the end of 2013 and for which irrigation was removed afterwards (irrigation stop).

The model was run for two scenarios for all three treatments (control, irrigation, irrigation stop) with the respective parameter sets. Scenario I mimics a tree with no ecological memory in which the model parameters ‘turnover rate of needles’, ‘turnover rate of sapwood’, and ‘turnover rate of carbon reserves’ were set to 1 yr (abbreviation ‘NoMemo’). Scenario II (abbreviation ‘Memo’) mimics a tree with more realistic turnover rates of needles (5 yr), sapwood (50) and carbon reserves (10) based on empirical findings. The number of Scots pine needle cohorts at Pfynwald was measured to be between three and five (data not shown). The average sapwood turnover rate was found to be between 45 and 55 yr (Fig. S2). Most uncertain was the estimation of the turnover rate of the carbon reserve (Gessler & Treydte, 2016). The metabolically active carbon reserve was reported to be 1–2 yr old (Gaudinski *et al.*, 2009), whereas the average age of all stem carbon reserves was found to be 10 yr (Carbone *et al.*, 2013). Richardson *et al*. (2015) explained this finding by the presence of two storage pools: a fast‐turning and a slow‐turning one. They assumed that the slow pool, however, was large enough that it cannot be ignored as a store of reserves and that it is over simplistic to assume a single pool that turns over quickly. As a consequence, we set the average turnover rate of the carbon pool to 10 yr.

### Statistical methods

SR data were aligned, cleaned and gap‐filled for gaps < 120 min with the R‐based (R Core Team, 2019) package treenetproc (Haeni *et al.*, 2020). Data gaps equal or longer than 120 min remained in the data set as NA.

The WF of the system‐analytic model were calculated in an optimisation process with Excel’s function Solver, maximising the determination coefficients for annual stem growth (GRO), needle (NL) and shoot length (SL) between consecutive years. These three variables were measured in the field as well as explicitly modelled and thus qualified to be used for the optimisation process. Parameterisation was run in three steps grouping WF according to their direct effect onto NL, SL and GRO. In the first step the two WF directly affecting NL were parameterised by optimising the determination coefficients between measured and modelled NL. In the second and third step, the same was performed with SL (2 WF) and GRO (11 WF).

The sensitivity of the model output to changes in the (set) turnover rates of needles, sapwood and carbon reserves was tested with a stepwise variation of each of the three turnover rates (Fig. S3). The average change in the output for annual stem growth was used as an indication for the model sensitivity to changes in the turnover rates. The turnover rate of carbon reserves had the lowest impact (weight 1) on model output, followed by sapwood turnover (2.3), and needle turnover had by far the highest impact on model output (2180) (Table S3).

The explanatory power of the simulations was quantified with the determination coefficient of linear regressions between measured and modelled means and standard errors of means per treatment.

## Results

### Soil water content

**Fig. 3:**
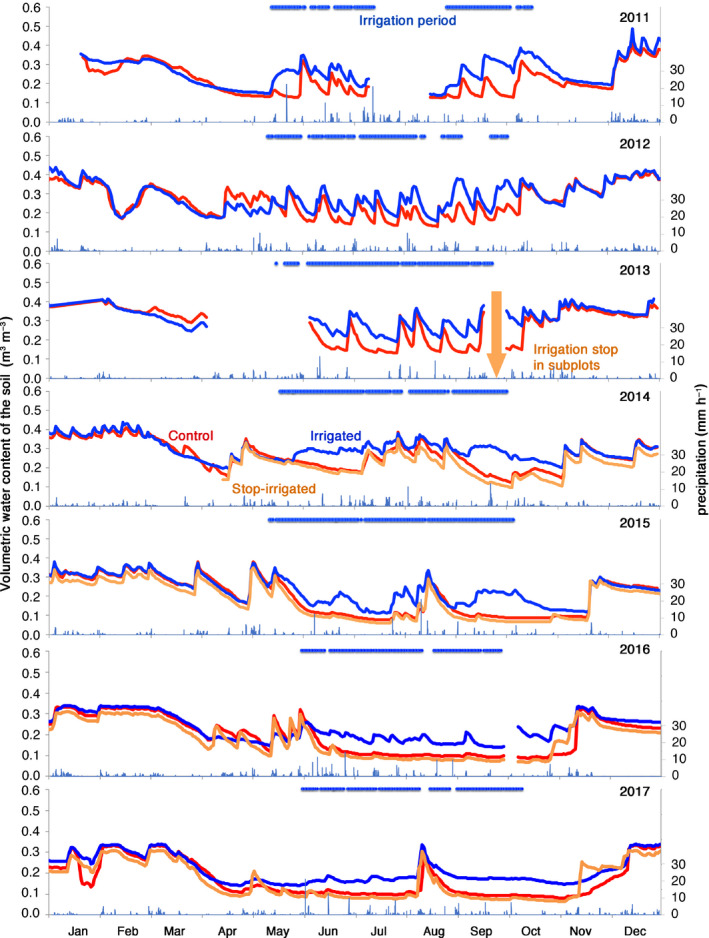
Mean daily time courses of soil volumetric water content (10–80 cm) in control plots (no irrigation, red), irrigated plots (blue) and plots where irrigation was stopped at the end of 2013 after 11 yr of treatment (orange). The irrigation was active during the nonfreezing period of the year. Periods of missing irrigation during the irrigation period indicate outages of the irrigation system (blue horizontal line). Data resolution: 1 h.

The irrigation treatment starting in 2003 considerably increased the soil water content of the treated plots (Fig. 3). However, with the stop of the irrigation in 2013, the level of soil water content (orange line) dropped to the same level as the control plot (red line) or even slightly below already in 2014. Generally, the water content of the soil with its low water holding capacity responded quickly to changes in the treatment, which became visible during periods of irrigation outages and during winter‐time when the irrigation was stopped.

### Tree water deficit

**Fig. 4:**
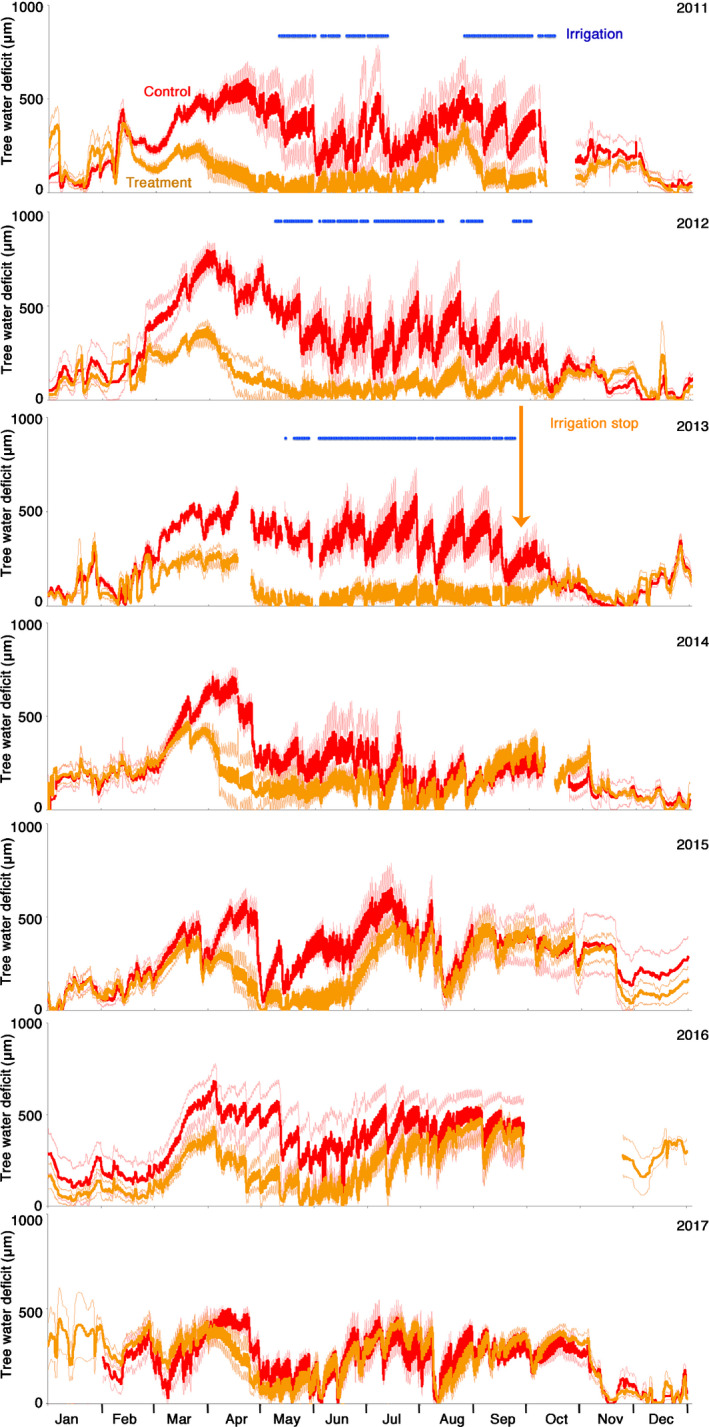
Time courses of tree water deficit (TWD) of irrigation stop (orange lines) and control trees (red lines) of Scots pine over 7 yr. At the end of 2013, the irrigation treatment (blue horizontal bars) was stopped. A TWD of zero means a fully hydrated tree. Increased TWD indicates stem shrinkage and thus an increased lack of water in the stem, meaning increased drought stress. Bold lines show the mean of three trees; the thin lines indicate the SE of the mean. Data resolution: 10 min.

The irrigation‐stop trees had a significantly lower tree water deficit (TWD) than the control trees during the irrigation period until the end of 2013 (Fig. 4). After switching off irrigation, the average annual TWD of the irrigation‐stop trees generally increased, however not linearly over the entire season. TWD remained reduced from March to July 2014–2016 (Fig. 4) before returning to the TWD level of the control trees during the later summer months. This intraseasonal pattern disappeared in the fourth year (2017) after the irrigation stop.

### Sap flow data

**Fig. 5:**
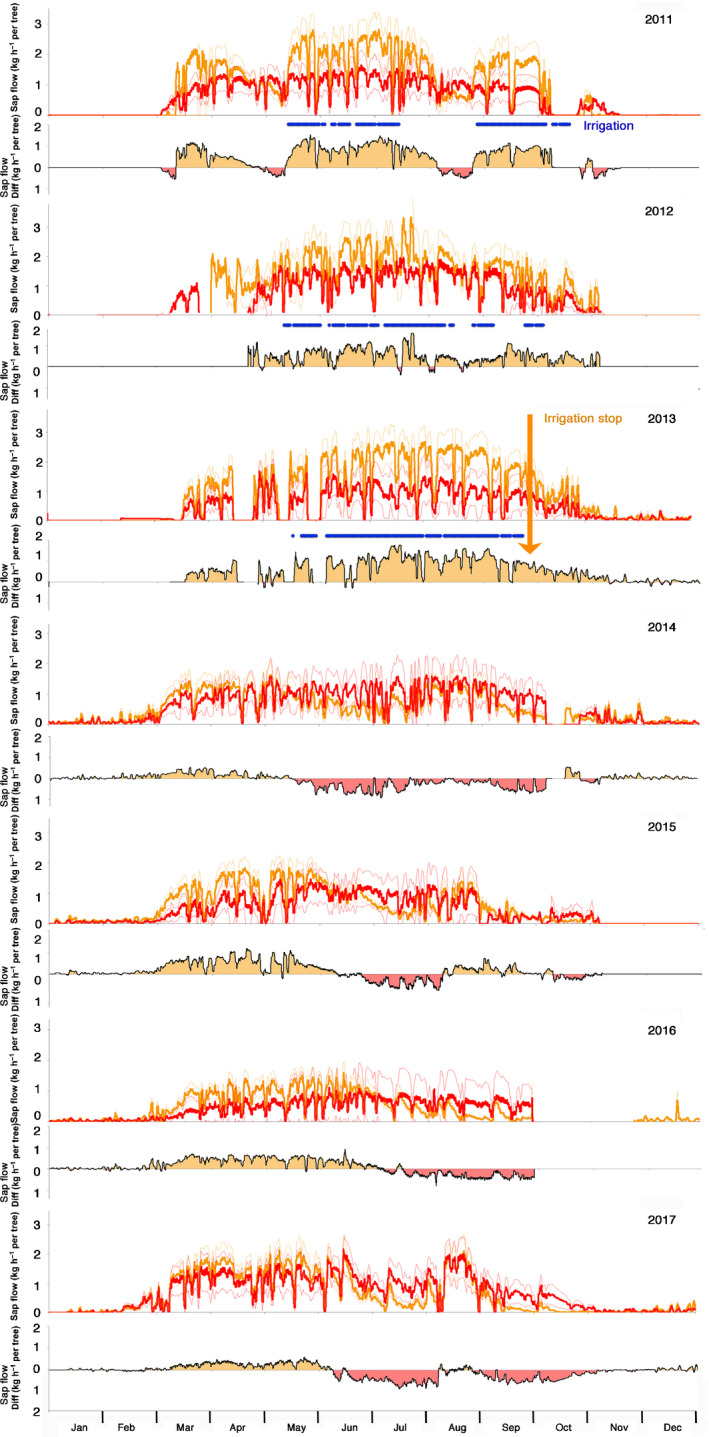
Running means of sap flow rates over 24 h of trees (*n* = 3) of the irrigation stop (orange) and the control (red) trees for the years 2011 to 2017 (Scots pine). Bold lines show the mean, the thin lines indicate the SE of the mean. Irrigation (blue horizontal line) was stopped at the end of 2013. The lower panel of each year shows the difference in sap flow rates between treated and control trees. Areas coloured in orange indicate higher sap flow rates of the treated trees, whereas areas coloured in red indicate higher sap flow rates of the control trees. Data resolution: 10 min.

Before stopping the irrigation treatment, sap flow rate of the irrigation‐stop trees was strongly increased during the summer months. During this time, sap flow reached values up to the double the rates of the control trees, however with a wide variation among individual trees (Fig. 5). During winter‐time, when the irrigation was stopped, both treatments behaved in a similar way. After the irrigation was stopped at the end of 2013, sap flow rates generally dropped significantly below the rates of the control trees during the summer months, but remained increased in spring when soil water availability was generally higher than in summer (Fig. 3). This pattern persisted over the entire measurement period until 2017. Interestingly, the short response time to reduced soil water availability became also visible during short‐term failures of the irrigation system (e.g. in July/August 2011, Fig. 5): as soon as the irrigation was stopped, the sap flow of the treated trees started to decrease and went markedly below the sap flow level of the control trees after *c*. 1–2 wk without irrigation. On a mean annual scale, sap flow remained downregulated below the level of the control trees for all 4 yr measured after stopping the irrigation.

### Radial stem growth

**Fig. 6:**
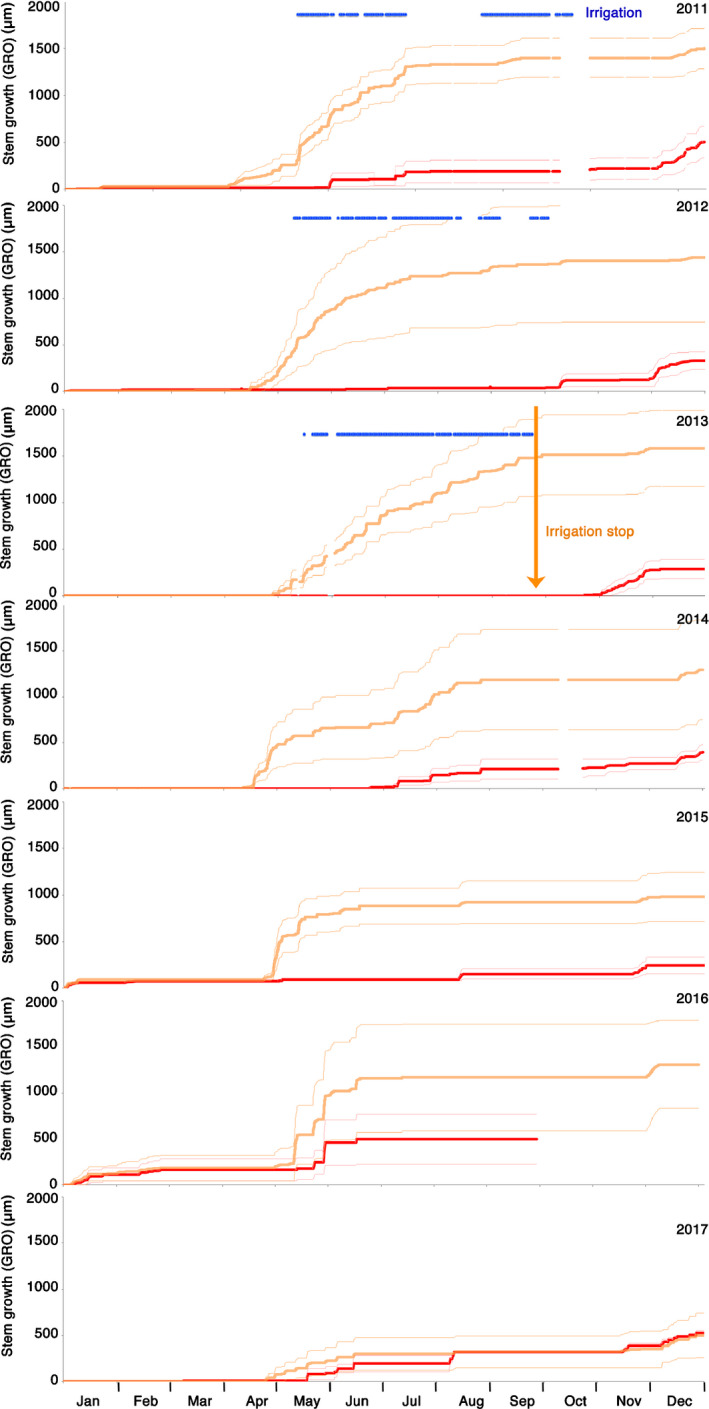
Mean radial stem increments (GRO) of wood and bark of Scots pine trees in control (no irrigation, red) and irrigation‐stop (orange) plots. Bold lines show the mean of three trees, the thin lines indicate the standard error of the mean. The irrigation (blue horizontal bars) was stopped at the end of 2013. The red line in 2016 ends prematurely because of a logger failure.

Radial stem growth (GRO) – deduced from SR measurement including irreversible bark and wood growth – was markedly increased in the irrigated compared with the control trees (Fig. 6). On average, the irrigated trees grew two to three times faster and started growth earlier than the control trees, however, with considerable individual differences. GRO decreased gradually after the irrigation stop but remained significantly above the growth rate of the control trees for three more years. In the fourth year after the irrigation was stopped (2017), the difference in GRO disappeared between the two treatments. An interesting detail is the often occurring GRO increase towards the end of the year which goes in parallel with the general soil rehydration at this time of the year (Fig. 3).

### Crown morphological measurements

**Fig. 7:**
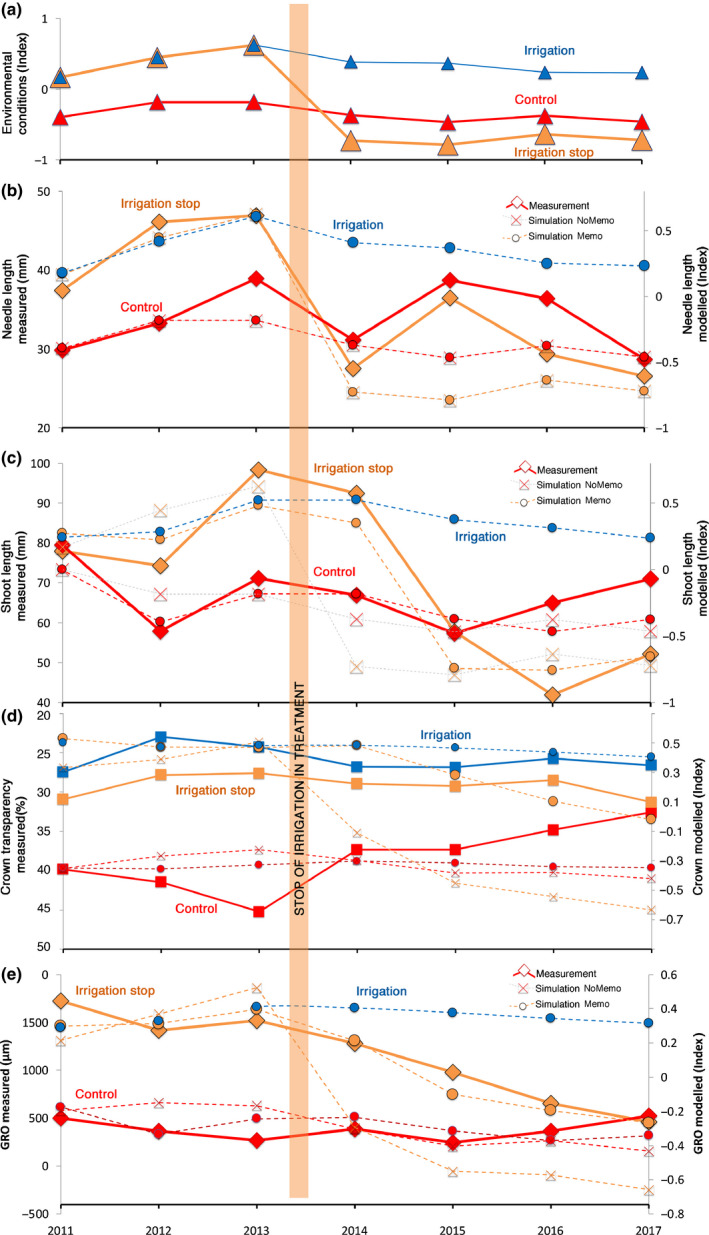
Measurements (full lines) vs model results (broken lines) of two scenarios ‘NoMemo’ (crossed squares) and ‘Memo’ (circles) of Scots pine. ‘NoMemo’ excluded any type of memory effects by setting the turnover rates of needles, sapwood and carbon reserves to 1 yr. Scenario ‘Memo’ set the turnover rates to more realistic values, that is 5 yr (needles), 50 yr (sapwood) and 10 yr (carbon reserves). (a) Environmental index (Supporting Information Methods S2) calculated for the control (red symbols), the irrigation stop (orange symbols) and the irrigated plots (blue symbols). Positive values indicate favourable growth conditions above the average, negative values indicate poor growth conditions below the average. (b) Measured and modelled needle lengths. (c) Measured and modelled shoot lengths. (d) Mean measured crown transparency and modelled crown status (proxy for leaf area). (e) Measured and modelled mean annual growth increments (GRO). Missing measurements or scenarios in some panels indicate not available data.

Needle length (NL) of the new cohort responded in the first year after the irrigation was stopped with a marked decrease (Fig. 7b). The needles grew even shorter than the ones of the control trees and kept this trend in the following years (Fig. 7b). SL remained high in the first year after stopping irrigation and strongly responded in the second year after the irrigation stop (Fig. 7c). SL remained markedly below the control in the following years.

Crown transparency (Fig. 7d) and GRO (Figs 6, 7e) showed the most distinct delays in their response. In both cases, the irrigation‐stop trees needed 3 to 4 yr to reach the same values as the control trees.

### Simulated tree responses

The simulations were run for all treatments (control, irrigation stop and irrigation) using the two alternative scenarios (Memo and NoMemo). The NoMemo scenario with a model parameterisation not allowing for legacy effects (Table S2) was not able to accurately simulate the measurements of the irrigation‐stop trees and led to a consistently lower explanatory power of the response variables SL and stem growth than the Memo scenario (Table 1). The only exception was with NL, which was insensitive to the turnover rates of needles, sapwood and carbon reserves and showed the same simulation output for both scenarios (Figs 7, S3; Tables 1, S3).

**Table 1:** Explanatory power of model simulations for measured variables stem growth (GRO), needle length (NL), and shoot length (SL) of Scots pine.

	Trees in control plot			Trees in irrigation‐stop plot			Trees in irrigated plot		
	*R* ^2^_NoMemo	*R* ^2^_Memo	Δ	*R* ^2^_NoMemo	*R* ^2^_Memo	Δ	*R* ^2^_NoMemo	*R* ^2^_Memo	Δ
Explanatory power of model for measurements									
GRO	19.2%	68.8%	49.6%	83.3%	97.1%	13.8%	na	na	na
NL	5.9%	5.9%	0.0%	79.2%	79.2%	0.0%	67.6%	67.6%	0.0%
SL	1.4%	16.2%	14.8%	34.7%	86.6%	52.0%	52.5%	87.5%	35.0%
Explanatory power of ENV for modelled variable GRO									
GRO	92.4%	19.8%	−72.7%	88.7%	56.9%	−31.8%	93.0%	63.1%	−29.9%

Listed are the determination coefficients of a linear regression (*R* ^2^) between the measured and modelled annual values for control, irrigation stop and permanently irrigated trees. Additionally, *R* ^2^ was calculated for the environmental index ENV and the modelled GRO. Δ is the difference between the two simulation scenarios ‘Memo’ and ‘NoMemo’. A positive value indicates an improvement of the explanatory power of the scenario ‘Memo’. na, not available.

By contrast, the Memo scenario with the more realistic turnover rates of needles (5 yr), sapwood (50) and carbon reserves (10) was able to predict the measured annual courses of radial stem growth and SLs significantly better for all treatments (control, irrigation stop and irrigation). The increased explanatory power of the Memo scenario compared with the NoMemo scenario ranged between 13.8% and 52%, depending on the variable (Table 1).

Contrary to the above described measurement–model relationships, the relationship between the environmental index ENV and the modelled radial stem growth (GRO) was stronger in the NoMemo scenario than in the Memo one (Table 1), showing that increasing the memory effect (by increasing the turnover rates) is reducing the responsiveness of GRO to current environmental conditions.

### Chronology of responses and the respective legacy effects

The irrigation‐stop treatment led to a reduction of the soil water content to a level comparable with – or even slightly lower than – the control plots (Fig. 3), and a cascade of tree physiological and morphological responses started (Figs 4, 5, 6, 7). On an annual timescale, NL, sap flow, and TWD responded already in the first year (Table 2). SL responded strongly in the second year, while radial stem growth and crown transparency did not respond abruptly but gradually, needing *c*. 4 yr to reach the level of the trees in the control plots (Fig. 7; Table 2).

**Table 2:** Responsiveness of annual means of Scots pine responses after stopping the irrigation.

	SAP	TWD	NL	SL	CT	GRO
First strong response (yr)	1	1	1	2	Gradual	Gradual
Matching control level (yr)	> 4	4	1	> 4	4	4
Legacy type	Negative (but see intraseasonal responses)	Negative (but see intraseasonal responses)	Negative	Negative with 1 yr delay	Positive	Positive

Listed are the number of years after the irrigation stop in 2013 when the measured variables returned to the level of the control trees. Measurements refer to sap flow (SAP), tree water deficit (TWD), needle length (NL), shoot length (SL), crown transparency (CT), and radial stem growth (GRO). CT and GRO did not show a strong response but were more of a gradual nature. The legacy type refers to the scheme in Fig. 1.

There appeared positive and negative legacy effects as shown in Fig. 1. Radial stem growth and crown transparency showed a distinct positive legacy effect (annual growth remaining above the control, crown transparency remaining below the control; Figs 6, 7), whereas the immediate biophysical responses related to tree water relations, that is sap flow (Fig. 5), TWD (Fig. 4) and also NL (Fig. 7), responded negatively (on a mean annual scale; Table 2) but with distinct intra‐annual deviations from the annual patterns (Figs 4, 5). Shoot length responded with a positive legacy effect in the first year after the irrigation stop (SL growth remained larger) and only negatively thereafter (shoots grew shorter than the control), that is, SL responded with a negative legacy effect with a 1‐yr delay.

## Discussion

### How to live with an overbuilt tree structure when soil water gets short?

The investigated pine trees grew larger and denser crowns during the 11 yr of irrigation compared with the control trees (Dobbertin *et al.*, 2010) and the functional structures such as sapwood (Schönbeck *et al.*, 2018) and roots (Brunner *et al.*, 2019) became adjusted to support this enlarged crown (Enquist & Niklas, 2002; Choat *et al.*, 2012). This response demonstrates the effect of water‐limited conditions on tree growth on the one hand, and the general tree response to a release from limiting conditions on the other hand, already documented several times also in other studies from this area (Feichtinger *et al.*, 2014; 2015; Herzog *et al.*, 2014; Grossiord *et al.*, 2018). The novel aspect here is how trees with an overbuilt structure deal with a water shortage after 11 well watered years. Are they able to benefit from potentially accumulated reserves or do they rather suffer from mal‐adjusted structures? In the following section, we discuss why both responses were found (positive and negative legacy effects; Figs 1, 7), depending on the organ or reserve that is considered (Table 2). With the help of the system‐analytical tree model, we further discuss why the turnover rates of organs and reserves are able to explain, at least partially, the different legacy effects (Fig. 7). Finally, we speculate what processes could explain the measured fact that irrigation‐stop trees grow better than the control trees despite the reduced soil water availability.

### Tree water relations – mostly negative legacy effects

The first response of a tree to the sudden reduction of water is of a biophysical nature. Drought‐stressed trees close their stomata and thus save water (Hetherington & Woodward, 2003; Zweifel *et al.*, 2012). The general reduction of the annual sap flow of the treated trees after the irrigation stop (Fig. 5) was thus most likely to be a consequence of the stomatal behaviour of an oversized transpiring area, suffering from a reduced water supply. On an annual timescale, the response of sap flow and TWD resembles a negative legacy effect (Fig. 1; Table 2), that is the downregulation of tree water relations is even stronger than the one observed for the control trees which grew under a continuous lack of water. However, there remained an important difference to this general annual pattern: during spring and early summer, sap flow remained higher (Fig. 5) and TWD remained lower (Fig. 4) than in the control trees. We hypothesise that the enlarged root biomass in the uppermost soil layer (Brunner *et al.*, 2019) might have led to an increased soil water uptake during the time when the general soil water availability was still high in this first phase of radial stem growth (Fig. 6). As a consequence, the soil dried out even faster than in the control plots (Fig. 3) and sap flow dropped in the second half of the summer below the rates of the control trees (Fig. 5). Less likely, but not excluded, is the possibility that deep roots tapped water in soil layers not covered by the soil water sensors. However, if this was the case, we expected a persistent positive effect on the sap flow, which was not measured.

### Needle and shoot lengths – negative legacy effects (with delay)

New built needles responded with an immediate length reduction in the year after the irrigation was stopped (negative legacy effect, that is needles grew shorter than the control), whereas the shoots responded 1 yr later (Fig. 7; Table 2, negative legacy effect with 1 yr delay), a phenomenon that has been reported for pine trees before (Dobbertin *et al.*, 2010; Feichtinger *et al.*, 2015). As an explanation for the NL reduction with drought, it has been proposed that the general decrease in leaf water potentials also reduces turgor pressure in the crown and thus, turgor pressure becomes limiting for needle growth (Myers, 1988; Giuggiola *et al.*, 2018; Guerin *et al.*, 2018). This lowered water potential was obviously not affecting the shoot growth at the same time (Table 2). The reason for that is not fully understood since both shoots and needles are predetermined in the buds built in the previous year (Chen *et al.*, 1996) and exposed to the same environmental conditions. However, the different responses of needles and shoots may be related to their tissue‐specific exposures to the low water potentials or eventually with tissue‐specific osmoregulation processes (Lazzarin *et al.*, 2019), as hypothesised also for radial stem growth (Coussement *et al.*, 2018). The model applied with the ‘Memo’ parameterisation (Fig. 7) – inducing a strong dependency of the shoot growth on conditions of the past year and thus reducing the dependency from current environmental conditions – was able to simulate the legacy effect with a 1‐yr delay. However, when cutting the functional link to the past year(s) – by setting the turnover rates of needles, sapwood and carbon reserves to 1 yr – the model was no longer able to simulate the measured shoot lengths accurately (Fig. 7; Table 1) and thus showing the importance of accounting for the turnover rates of organs and carbon reserve in the model to explain the trees’ lagged response to current conditions.

### Radial stem growth and total leaf area: positive legacy effects

After stopping irrigation, annual stem growth and total leaf area (as indicated by measured crown transparency data) clearly remained above the level of the control trees for 3 more years before matching the control level (Fig. 7). This is assigned to a positive legacy effect (Fig. 1) and indicates that this high growth rate cannot be explained by current environmental conditions alone but is positively influenced by the past, more favourable, conditions. The ‘Memo’ model balanced the status of the accumulated carbon reserves and particularly the enlarged total leaf area – two positive effects – against the direct impact of the environment (negative effect) on radial stem growth (Table S2). The simulation results support this statement by demonstrating that the ‘Memo’ model (Table S2) is able to simulate the observed growth pattern always better than can the ‘NoMemo’ model (Fig. 7; Table 1).

### Turnover rates of organs and reserves determine legacy effects

The turnover rate of needles of the pine trees was measured to be 3–5 yr at our site, corresponding with the legacy effect in crown transparency and radial stem growth which lasted *c*. 4 yr. The ‘Memo’ parameterisation of the model assumed a turnover rate for the needles of 5 yr (which indicates that only *c*. 20% of the total leaf area is replaced every year) and this model parameter (Table S2) was found to be crucial to be able to track the observed annual stem growth dynamics.

Interestingly, a sensitivity analysis of the ‘Memo’ model gave the highest correlation between model output and measured radial stem growth data when a needle turnover rate of 3 yr was used (Fig. S3). This implies that the initially set needle turnover rate of 5 yr could be adjusted to 3 yr in order to further increase the goodness‐of‐fit. Furthermore, the turnover rates of sapwood and carbon reserves contributed to the modelling quality, however with much lower weights (Table S3).

According to our crown transparency measurements (Fig. 7), the irrigation‐stop pines took *c*. 4 yr to adjust their leaf area to the new conditions. Furthermore, the simulation of the crown status (proxy for total leaf area) indicated that the adjustment time might even be longer, as crown status remained still increased after 4 yr (of modelling) (Fig. 7). Additionally, the lag of 4 yr matched exactly the time during which measured radial stem growth rates appeared to be increased in relation to the expected values from the control trees, and it was also about the duration in which the needle biomass was totally renewed (needle lifetime was measured to be between 3 and 5 yr). An argumentation that sets these facts into a causal relationship as carried out in our model, intrinsically implies a partial decoupling of growth from current environmental conditions as clearly supported by our results (Table 1).

### Leaf area determines radial stem growth

‘The resources grow the leaves and the leaves grow the tree’ is an old saying of some foresters and which gets reappraised with this study. The rapid response of the needle growth to the current conditions fits the first part of the statement, whereas the delayed stem growth response supports the second part. Physiologically, we argue that the enlarged leaf area of the irrigation‐stop trees demands an adequate water supply system that can support the high potential transpiration (Anfodillo *et al.*, 2016). In other words, the sapwood area (particularly the annual sapwood increment) is needed to support the potential evaporative demand of the crown. This balanced relationship between leaves and their amount of xylem (Martinez‐Vilalta *et al.*, 2009) requires a corresponding amount of xylem at the stem level, also well known as pipe model theory, which basically assumes a distinct number of xylem conduits per leaf area (Shinozaki *et al.*, 1964; Mencuccini & Grace, 1996; Sterck & Zweifel, 2016). As a consequence, we assume a direct physiological causality between leaf area and wood growth (Zweifel *et al.*, 2006; Fatichi *et al.*, 2014; Zweifel & Sterck, 2018), which can lead to an increased radial stem growth despite drought. However, what mechanism is able to explain that?

### Radial stem growth despite drought stress: towards an explanation

We propose two speculative explanations for the increased radial stem growth rates despite the drought stress conditions in the irrigation‐stop treatment: (1) the enlarged root biomass (Herzog *et al.*, 2014; Brunner *et al.*, 2019) improved the water uptake capacity, and (2) osmoregulation actively increased turgor pressure in the cambium in order to reach the threshold for cell growth (Hsiao & Xu, 2000; Coussement *et al.*, 2018). The first point (1) is discussed in the second paragraph of the Discussion and could explain the more efficient water uptake with higher sap flow rates and lower tree water deficits allowing these trees to keep cellular turgor pressure high enough to allow for cell division and cell enlargement (Lockhart, 1965; Petit *et al.*, 2011; Ortega *et al.*, 2012). (2) A second effect we speculate about here is related to cellular osmoregulation in the bark including the cambium (e.g. O'Brien *et al.*, 2014; Lintunen *et al.*, 2016) implying an active investment in growth during drought. Turgor pressure in living tissues is not only determined by physical conditions, that is the dryness of air and soil, but is also affected by active biological processes such as the increase of the osmotic potential in cells by sugar loading (Badalotti *et al.*, 2000; De Schepper & Steppe, 2010, 2011; Barraclough *et al.*, 2018, 2019a,b; Lazzarin *et al.*, 2019; Michelot‐Antalik *et al.*, 2019). We speculate that the irrigation‐stop trees avoided the low turgor pressure in the cambium with the mobilisation of osmotically active compounds. Recent work at the same research site showed, indeed, that such active osmoregulation might take place in pine trees (Mencuccini *et al.*, 2017; Lazzarin *et al.*, 2019). However, it was not particularly measured for the irrigation‐stop trees and the osmoregulation effect was mainly measured in the branches and not in the stem (Lazzarin *et al.*, 2019).

Proposing an osmoregulation mechanism also raises the question whether these additional resources for keeping high growth rates are available and for how long they could last. There is evidence that the irrigated trees had more reserves available than the control trees. A recent study of pine trees at the same site showed an overall increase in nonstructural carbohydrates (NSC) in the stems of irrigated trees (von Arx *et al.*, 2017) and Schönbeck *et al*. (2018) found stem growth and NSC being positively related to total leaf area, which was larger for the irrigated trees compared with the control. We hypothesise that the irrigation‐stop trees relied on the additional carbon reserves and kept the reduction of available energy for extra growth under drought conditions in balance with the rate of reduction of the leaf area. As long as a tree is able to keep its radial stem growth high with an extra investment of energy to support the large crown, it may be able to reduce its crown size slowly. Obviously, no reserve is infinite and becomes exhausted eventually. At this time, the tree’s leaf area should be reduced to a size that is in balance with the new dry conditions.

### Conclusions

The sudden reduction in soil water availability after the irrigation stop did not lead to a rapid decrease in radial stem growth of pine trees, as we expected. Instead, radial stem growth was found to be in line with a slow reduction of the leaf area, taking 4 yr of time to reach the level of the never irrigated control trees. From a functional point of view, we conclude that leaf area imposes radial stem growth in order to keep the balance between transpiring surface and supporting sapwood.

The modelling results suggest turnover rates of organs and carbon reserve as important determinants of legacy effects on trees. Particularly, crown size and stem growth seem to be strongly determined by past conditions and processes due to the needle lifetime of *c*. 4 yr, affecting the direct growth response to current environmental conditions. In other words, we showed that the biological predisposition of a pine tree is able to strongly decouple growth from current environmental conditions. Further we propose that an osmoregulation mechanism may help to explain the increased radial stem growth despite the suddenly reduced availability of soil water. Future work is called to particularly focus on the species‐specific aspects of these findings.

## Author contributions

RZ (idea, paper concept, data acquisition, data analyses, modelling, interpretation of results, writing); SE (data analyses, interpretation of results, writing); FS (modelling, interpretation of results, writing); AG (interpretation of results, writing); TA (interpretation of results, writing); MM (interpretation of results, writing); GvA (data acquisition, interpretation of results, writing); ML (data acquisition, interpretation of results, writing); MH (data acquisition, data analyses, interpretation of results, writing); LF (data acquisition, data analyses, interpretation of results, writing); KM (data acquisition, data analyses, interpretation of results, writing); SK (data analyses, interpretation of results, writing); LW (interpretation of results, writing); YS (interpretation of results, writing); AKB (interpretation of results, writing); LS (interpretation of results, writing); CH (data acquisition, interpretation of results, writing); NDG (data acquisition, interpretation of results, writing); AG (data acquisition, interpretation of results, writing); MS (fund rising infrastructure, interpretation of results, writing); AR (idea research infrastructure, research coordination, fund rising infrastructure, interpretation of results, writing).

## Supporting information


**Fig. S1** Distribution of annual precipitation over Switzerland and the location of the research site Pfynwald.
**Fig. S2** Frequency distribution of sapwood rings per tree and sapwood widths.
**Fig. S3** Sensitivity of model output to changes in the turnover rates of needles, sapwood and carbon reserves.
**Methods S1** Model equations.
**Methods S2** Calculation of environmental index.
**Table S1** Diameter at breast height and tree height for the pine (*Pinus sylvestris*) trees equipped with dendrometer and sap flow sensors and group into subplots with treatment.
**Table S2** Model parameters and their values for the three different treatments and the two scenarios.
**Table S3** Sensitivity analyses of model output to changes of the turnover rates of needles, sapwood and carbon reserves.
Please note: Wiley Blackwell are not responsible for the content or functionality of any Supporting Information supplied by the authors. Any queries (other than missing material) should be directed to the *New Phytologist* Central Office.
