# Supplementary material for: Determinants of legacy effects in pine trees – implications from an irrigation‐stop experiment
Source: New Phytol. 2020 May 9;227(4):1081–96. doi: 10.1111/nph.16582 (PMC7383578; doi:10.1111/nph.16582)
Supplement: Supplementary file 1 — Fig. S1 Distribution of annual precipitation over Switzerland and the location of the research site Pfynwald. Fig. S2 Frequency distribution of sapwood rings per tree and sapwood widths. Fig. S3 Sensitivity of model output to changes in the turnover rates of needles, sapwood and carbon reserves. Methods S1 Model equations. Methods S2 Calculation of environmental index. Table S1 Diameter at breast height and tree height for the pine (Pinus sylvestris) trees equipped with dendrometer and sap flow sensors and group into subplots with treatment. Table S2 Model parameters and their values for the three different treatments and the two scenarios. Table S3 Sensitivity analyses of model output to changes of the turnover rates of needles, sapwood and carbon reserves. Please note: Wiley Blackwell are not responsible for the content or functionality of any Supporting Information supplied by the authors. Any queries (other than missing material) should be directed to the New Phytologist Central Office. [file NPH-227-1081-s001.pdf]

New Phytologist

***New Phytologist* Supporting Information**

Article title: **Determinants of legacy effects in pine trees - implications from an irrigation-stop experiment**

Authors: Roman Zweifel, Sophia Etzold, Frank Sterck, Arthur Gessler, Tommaso Anfodillo, Maurizio Mencuccini, Georg von Arx, Martina Lazzarin, Matthias Haeni, Linda Feichtinger, Katrin Meusburger, Simon Knuesel, Lorenz Walthert, Yann Salmon, Arun K. Bose, Leonie Schoenbeck, Christian Hug, Nicolas De Girardi, Arnaud Giuggiola, Marcus Schaub, Andreas Rigling

Article acceptance date: 24 March 2020

The following Supporting Information is available for this article:

Fig. S1 Distribution of annual precipitation over Switzerland and the location of the research site Pfynwald.

Fig. S2 Frequency distribution of sapwood rings per tree and sapwood widths.

Fig. S3 Sensitivity of model output to changes in the turnover rates of needles, sapwood, and carbon reserves.

Table S1 Diameter at breast height and tree height for the pine (Pinus sylvestris) trees equipped with dendrometer and sap flow sensors and group into subplots with treatment.

Table S2 Model parameters and their values for the three different treatments and the two scenarios.

Table S3 Sensitivity analyses of model output to changes of the turnover rates of needles, sapwood and carbon reserves.

Methods S1 Model equations.

Methods S2 Calculation of environmental index.

Fig. S1 Distribution of annual precipitation over Switzerland. The research site Pfynwald is located in one of the driest regions of the Alps, with about 550 mm precipitation per year. (Source: Dirk Schmatz, WSL).

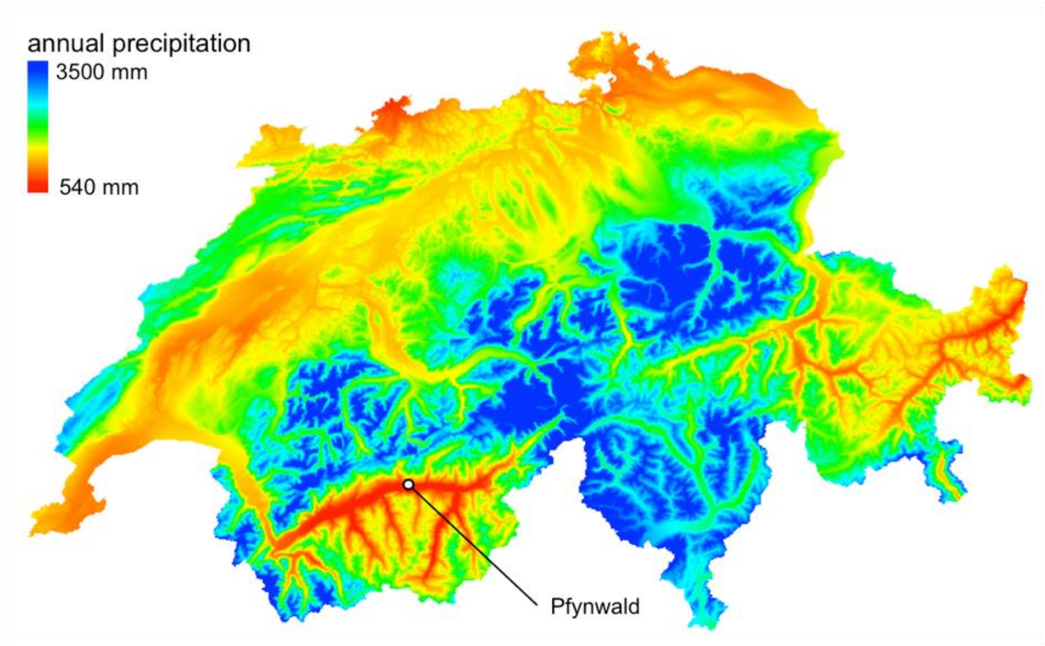

Fig. S2 Frequency distribution of sapwood rings per tree and sapwood widths. Measured were 20 trees in the control plots and 20 trees in the irrigated plots in the year 2013. Mean +- standard deviation is given in the panel titles. The numbers in the bars indicate the number per group. The values in brackets [years] indicate the borders of the four groups.

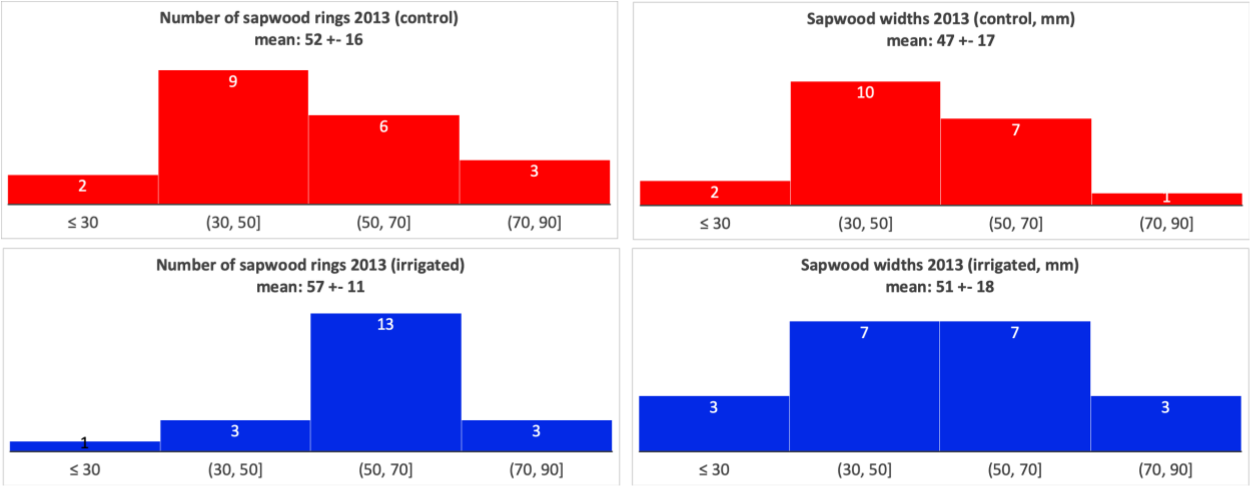

Fig. S3 Sensitivity of model output to changes in the turnover rates of needles, sapwood, and carbon reserves. **a)** Measured and modelled time series of annual stem growth (GRO). The lines show the modelled GRO with different needle turnover rates varying between 1 and 10 years (bold line = 5). **b)** Changes of the R^2 between measured and modelled GRO when varying the respective turnover rates. **c)** Changes of the R^2 between measured and modelled needle length (NL) when varying the respective turnover rates. **d)** Changes of the R^2 between measured and modelled shoot length (SL) when varying the respective turnover rates.

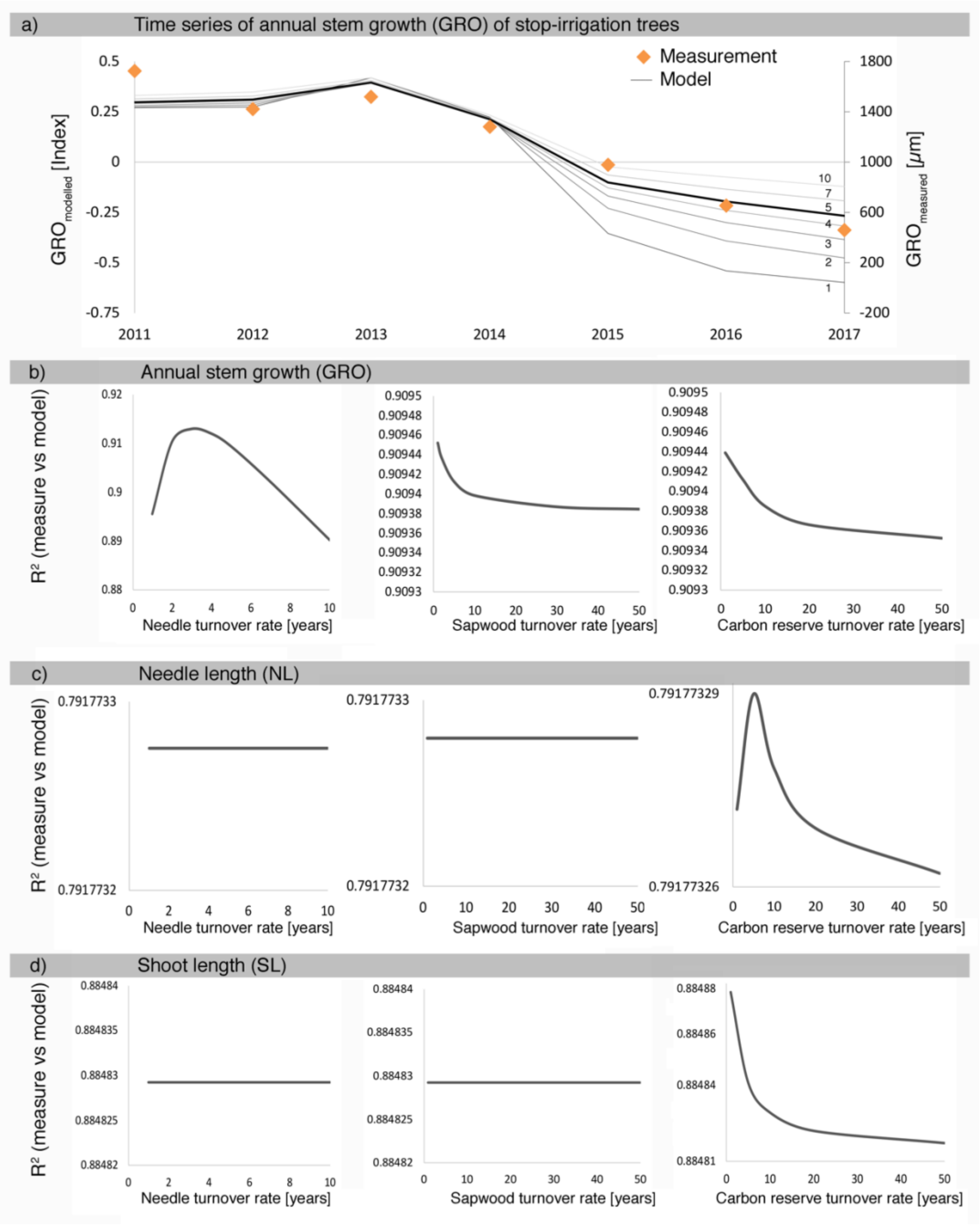

Table S1 Diameter at breast height (DBH in cm) and tree height (Tree_height in m) for the pine (Pinus sylvestris) trees (Tree_ID) equipped with dendrometer and sap flow sensors and group into subplots with treatment. Control_P1: not irrigated, Irr_Stop_P2: irrigated from 2003 until 2013 and cut off from irrigation in the following years, Irrigation_P2: irrigated.

Tree_ID	Site_subplot	Tree_DBH	Tree_height
110	Control_P1	22.5	12.8
124	Control_P1	29.2	11.8
109	Control_P1	20.7	11.2
274	Irr_Stop_P2	24.4	12.1
276	Irr_Stop_P2	32	13.0
275	Irr_Stop_P2	22.7	11.5
247	Irrigation_P2	19.2	12.6
246	Irrigation_P2	24.2	12.2
250	Irrigation_P2	29.4	13.4

Table S2 Model parameters and their values for the three different treatments (Ctr = never irrigated control plots, StopIrr = irrigation stop in 2013, Irr = irrigated plots) and two scenarios (NoMemo = turnover (TO) rates set to 1 year, Memo = TO rates set realistic values). TO rates are pre-set parameters. The weighting factors (WFx) are optimized parameters. The optimisation process was forced to keep the parameters within the range from 0.1 to 100. The shaded values were set to zero in order to cut the legacy effect in the NoMemo scenario. The broken horizontal lines indicate the three groups of parameters used for the model optimization process.

**Treatment**<br>**Scenario**	Ctr<br>Memo	Ctr<br>NoMemo	StopIrr<br>Memo	StopIrr<br>NoMemo	Irr<br>Memo	Irr<br>NoMemo
**Pre-set parameters**						
TO rate needles	5	1	5	1	5	1
TO rate sapwood	50	1	50	1	50	1
TO rate C-pool	10	1	10	1	10	1
**Optimized parameters**						
WF_1 needle length: ENV	0.2	0.2	100.0	100.0	100.0	100.0
WF_2 needle length: bud status	0.1	0.1	0.1	0.1	0.1	0.1
WF_3 shoot length growth: ENV	0.1	0.1	2.1	2.1	11.5	11.5
WF_4 shoot length growth : C-pool status	100.0	0.0	41.3	0.0	9.6	0.0
WF_5 crown growth: needle growth	0.1	0.1	0.1	0.1	0.1	0.1
WF_6 crown growth: shoot growth	100.0	100.0	100.0	100.0	100.0	100.0
WF_7 C-pool growth: ENV	0.1	0.1	59.4	59.4	59.3	59.3
WF_8 C-pool growth: crown growth	8.0	8.0	0.1	0.1	0.1	0.1
WF_9 stem growth: ENV	1.9	1.9	9.0	9.0	16.9	16.9
WF_{10} stem growth: crown status	6.0	6.0	100.0	100.0	100.0	100.0
WF_{11} stem growth: crown growth	0.1	0.1	0.3	0.3	22.7	22.7
WF_{12} stem growth: C-pool status	10.9	0.0	0.1	0.0	0.1	0.0
WF_{13} stem growth: sapwood status	0.1	0.1	0.1	0.1	0.1	0.1
WF_{14} bud status: ENV	100.0	100.0	99.9	99.9	100.0	100.0
WF_{15} bud status: C-Pool status	0.1	0.0	0.1	0.0	0.1	0.0

Table S3 Sensitivity analyses of the model output variables stem growth (GRO), needle length (NL), and shoot length (SL) to changes of the parameters 'turnover (TO) rates of needles', 'TO rates of sapwood' and 'TO rates of carbon pool'. The presented results refer to the stop irrigation treatment with the scenario Memo. GRO was most sensitive to the TO rate of needles. NL was not affected by any of the TO parameters since NL growth was almost only driven by the environmental index ENV. SL responded most sensitive to changes of the TO rate of the carbon reserve (C-pool).

**Effect on:**	**GRO**	**NL**	**SL**
**TO rate needles**	2180	no effect	no effect
**TO rate sapwood**	2.3	no effect	no effect
**TO rate C-pool**	1	no effect	1

Methods S1 Model equations.

Model input

The model input is an index value between -1 (poor) and +1 (rich) corresponding to the environmental condition (ENV). The calculation of ENV is described in Methods S2.

Needle length growth (NL) (arrows 1 and 2 in Fig. 2)

$$NL = (ENV*WF_1 + BS*WF_2)/(WF_1+WF_2) \quad \text{Eq. 1}$$

where *NL* is needle length growth, *BS* is the bud status before the new growing period, *ENV* is the environmental condition, and WF_1 and WF_2 are the respective weighting factors.

Shoot length growth (SL) (arrows 3 and 4 in Fig. 2)

$$SL = (ENV*WF_3 + BS*WF_4)/(WF_3+WF_4) \quad \text{Eq. 2}$$

where *SL* is shoot length growth, *BS* is the bud status before the new growing period, *ENV* is the environmental condition, and WF_3 and WF_4 are the respective weighting factors.

Crown growth (CG) (arrow 5 in Fig. 2)

$$CG = (NL*WF_5 + SL*WF_6)/(WF_5+WF_6) \quad \text{Eq. 3}$$

where *CG* is crown growth, and WF_5 and WF_6 are the respective weighting factors.

Carbon reserve growth (CRG) (arrows 6 and 7)

$$CRG = (ENV*WF_7 + CG * WF_8)/(WF_7 + WF_8) \quad \text{Eq. 4}$$

where *CRG* is the growth in carbon reserves, and WF_7 and WF_8 are the according weighting factors.

Stem growth (SG) (arrows 8 to 12)

$$SG = (ENV*WF_9 + CS*WF_{10} + CG*WF_{11} + CRS_{old}*WF_{12} + SS_{old}\ WF_{13})/(WF_9 + WF_{10} + WF_{11} + WF_{12} + WF_{13}) \quad \text{Eq. 5}$$

where SG is stem growth, CRS_{old} is the carbon reserves status during the past dormancy period, SS_{old} is the sapwood status of the past dormancy, and WF_9 to WF_{13} are the according weighting factors.

Crown status (CS) (arrows 13 and 14)

$$CS_{new} = (CS_{old} * TO\_rate_{needles} + CG)/(1 + TO\_rate_{needles}) \qquad \text{Eq. 6}$$

where CS_{new} is the new crown status after the growing period, CS_{old} is the old crown status before the growing period, and $TO\_rate_{needles}$ quantifies the turnover rate of the needles.

Bud status (BS) (arrows 15 and 16)

$$BS_{new} = (ENV * WF_{14} + CRS_{new} * WF_{15})/(WF_{14} + WF_{15}) \qquad \text{Eq. 7}$$

where BS_{new} is the new bud status after the growing period, CRS_{new} is the current carbon reserve status and WF_{14} and WF_{15} are the respective weighting factor.

Carbon reserve status (CRS) (arrows 17 and 18)

$$CRS_{new} = (CG + CRS_{old} * TO\_rate_{carbon})/(1 + TO\_rate_{carbon}) \qquad \text{Eq. 8}$$

where CRS_{new} is the new carbon reserve status after the growth period, and TO_rate_{carbon} is the turnover rate of the carbon reserves.

Sapwood status (SS) (arrows 19 and 20)

$$SS_{new} = (CRG + SS_{old} * TO\_rate_{sapwood})/(1 + TO\_rate_{sapwood}) \qquad \text{Eq. 9}$$

where SS_{new} is the new sapwood status after the growing period, and $TO\_rate_{sapwood}$ is the lifespan of the sapwood.

Methods S2 Calculation of environmental index

$$Index_{Soil} = \frac{VWC_y}{\frac{1}{n}\sum_{i=1}^{n} VWC\_ctr\_irr_i} - 1 \qquad \text{Eq. 10}$$

where *Index*$_{soil}$ is the index of the volumetric soil water content (VWC) in the year y relative to the mean VWC of control (ctr) and irrigated (irr) plots of a reference period n. The index ranges from -1 to +1.

$$Index_{PrercipIrr} = \frac{PrecipIrr_y}{\frac{1}{n}\sum_{i=1}^{n} PrecipIrr\_ctr\_irr_i} - 1 \qquad \text{Eq. 11}$$

where *Index*$_{PrecipIrr}$ is the index of the precipitation (Precip) including the additionally irrigated water (Irr) in the year y relative to the mean value in control (ctr) and irrigated (irr) plots of a reference period n. The index ranges from -1 to +1.

$$Index_{TempRad} = \frac{\left(2 - \frac{Temp_y}{\frac{1}{n}\sum_{i=1}^{n} Temp_i} + \frac{Rad_y}{\frac{1}{n}\sum_{i=1}^{n} Rad_i}\right)}{2} - 1 \qquad \text{Eq. 12}$$

where *Index*$_{TempRad}$ is the index of temperature (Temp) and net radiation (Rad) in the year y relative to the mean value of a reference period n. The index ranges from -1 to +1.

$$Index_{ENV} = \frac{Index_{soil} + Index_{PrecipIrr} + Index_{TempRad}}{3} - 1 \qquad \text{Eq. 13}$$

where *Index*$_{ENV}$ is the environmental (ENV) index averaging the indices from Eq. 10-12. The index can range from -1 to +1 and was used as input for the model (Fig. 2).

References S Click here to enter text.

Dengler NG. 2001. Regulation of vascular development. *Journal of Plant Growth Regulation* **20**: 1-13.

Gessler A, Treydte K. 2016. The fate and age of carbon - insights into the storage and remobilization dynamics in trees. *New Phytologist* **209**(4): 1338-1340.

Hoch G, Richter A, Körner C. 2003. Non-structural carbon compounds in temperate forest trees. *Plant, Cell and Environment* **26**: 1067-1081.

Zweifel R, Sterck F. 2018. A conceptual tree model explaining legacy effects on stem growth. *Frontiers in Plant Sciences* **1**(9): 1-9.

Zweifel R, Zeugin F, Zimmermann L, Newbery DM. 2006. Intra-annual radial growth and water relations of trees – implications towards a growth mechanism. *Journal of Experimental Botany* **57**(6): 1445-1459.
